# Increased retention of tau PET ligand [^18^F]-AV1451 in Alzheimer’s Disease Psychosis

**DOI:** 10.1038/s41398-022-01850-z

**Published:** 2022-02-26

**Authors:** J. J. Gomar, G. Tan, J. Halpern, M. L. Gordon, B. Greenwald, J. Koppel

**Affiliations:** 1 Feinstein Institutes for Medical Research, Manhassett, NY USA; 2 Department of Psychiatry, Zucker Hillside Hospital, Northwell Health, Glen Oaks, NY USA

**Keywords:** Biomarkers, Neuroscience

## Abstract

Psychosis in Alzheimer’s disease (AD) represents a distinct disease subtype with a more rapid progression of illness evidenced by an increased velocity of cognitive decline and a hastened mortality. Previous biomarker and post-mortem studies have implicated tau neuropathology as a possible mediator of the accelerated decline in AD psychosis. Tau positron emission tomography (PET) neuroimaging provides the opportunity to evaluate tau pathology in-vivo, so that clinical symptomatology can be correlated with disease pathology. [^18^F]-AV1451 (Flortaucipir) is a PET ligand with high affinity for insoluble paired-helical filaments (PHFs) of hyperphosphorylated tau. In order to determine whether the development of psychosis and worsened prognosis in AD is associated with an increased burden of tau pathology that can be identified with tau imaging, we identified subjects within the Alzheimer’s disease neuroimaging initiative (ADNI) who had [^18^F]-AV1451 imaging at baseline and became psychotic over the course of the study (*N* = 17) and matched them 1:3 for gender, age, and education to subjects who had [^18^F]-AV1451 imaging at baseline and did not become psychotic (*N* = 50). We compared baseline [^18^F]-AV1451 retention, in addition to cognitive and functional baseline and longitudinal change, in those who became psychotic over the course of participation in ADNI with those who did not. Results suggest that increases in tau pathology in frontal, medial temporal, and occipital cortices, visualized with [^18^F]-AV1451 binding, are associated with psychosis and a more rapid cognitive and functional decline.

## Introduction

Psychosis occurring over the course of Alzheimer’s disease (AD), comprising the idiopathic development of fixed false beliefs or disturbances of perception [1], represents a distinct phenotype [2] that is a harbinger of an accelerated velocity of cognitive and functional decline leading to hastened mortality [3–8]. Psychosis in AD, which has been reported to have a cross-sectional prevalence of 40% [9], adds to caregiver burden [10] as it is often complicated by agitation and aggression [1]. The challenge of coping with and responding to a loved one under the influence of psychotic symptoms is a common precipitant of long-term care placement [11]. To date, there are no medications that are approved by FDA to treat psychotic symptoms in dementia, and although antipsychotics have demonstrated efficacy, they also carry a black box warning from FDA owing to an increase in mortality associated with their use [12].

The accelerated trajectory of cognitive impairment that runs tandem with the development of psychosis in AD may be related to a more rapid accrual of neuropathology that disrupts neural networks involved in reasoning and perception. Of the abnormally aggregated microtubule associated tau and amyloid-beta (Aβ) protein pathologies implicated in AD, only the locations of neurofibrillary tangles comprising hyperphosphorylated tau correlate topographically with the particular domains of cognitive dysfunction [13–15]. Similarly, studies of the neuropathologic correlates of psychosis in AD also suggest a specific relationship with tau pathology. Perturbations in cerebrospinal fluid (CSF) levels of tau rather than amyloid previously associated with an increased rate of cognitive decline [16] have also been reported in patients with psychotic AD [17]. In post-mortem studies, increased tangle pathology [18–20] and an excess of phosphorylated tau [21, 22] have been associated with a history of psychosis. Taken together, these studies suggest that an excess burden of tau pathology may be a risk factor for the development of psychosis, and may partially explain its more rapid cognitive decline. To date, no published studies have utilized tau neuroimaging to study the in-vivo relationship between tau pathology and the development of psychotic symptoms in AD.

[^18^F]-AV1451 (Flortaucipir) is a PET ligand with high affinity for insoluble paired-helical filaments (PHFs) of hyperphosphorylated tau [23]. Mirroring the relationship between neurofibrillary tangle pathology and clinical disease severity, [^18^F]-AV1451 binding patterns increase in the presence of cognitive impairment and are topographically associated with cortical atrophy and clinical symptomatology [24, 25]. Previously published results from ADNI suggest that [^18^F]-AV1451 retention discriminates AD from mild cognitive impairment (MCI) and healthy controls, and correlates with CSF tau biomarkers [26].

We have previously reported on an accelerated cognitive decline [8]; regional metabolic deficits identified with FDG-PET [8]; and elevations in CSF tau [17] in ADNI participants who developed psychosis over the course of study follow-up. In order to determine whether the development of psychosis and worsened prognosis in AD is associated with an increased burden of tau pathology that can be identified with tau imaging, we compared baseline [^18^F]-AV1451 retention together with cognitive and functional decline in those who became psychotic over the course of participation in ADNI with those who did not. Results suggest that increases in tau pathology, visualized with [^18^F]-AV1451, are associated with psychosis and a more rapid cognitive and functional decline.

## Materials and methods

### Participants

Data used in the preparation of this article were obtained from the Alzheimer’s Disease Neuroimaging Initiative (ADNI) database (adni.loni.usc.edu). The ADNI was launched in 2003 as a public-private partnership, led by Principal Investigator Michael W. Weiner, MD. The primary goal of ADNI has been to test whether serial magnetic resonance imaging (MRI), positron emission tomography (PET), other biological markers, and clinical and neuropsychological assessment can be combined to measure the progression of mild cognitive impairment (MCI) and early Alzheimer’s disease (AD).

We examined the ADNI database to explore the association of tau burden in those AD participants who became psychotic (AD + P) compared to those who did not (AD − P) over the course of participation in ADNI, using PET imaging with the radiotracer [18F]-AV1451. The presence/absence of psychotic symptoms was assessed utilizing the first 2 items of the 12-item Neuropsychiatric Inventory (NPI-Q) [27], i.e. delusions and hallucinations; as in previously published ADNI analyses [8, 17, 28], and consistent with consensus criteria for psychosis in dementia [1]. ADNI participants were classified as AD + P if they had a score >0 on either of those items over the course of the study while AD − P participants maintained a score of 0 on each. We identified 17 AD + P study participants with a valid [^18^F]-AV1451 PET scan. From a pool of 118 AD − P subjects in the study with a valid [^18^F]-AV1451 PET scan, subjects in the AD + P cohort were matched for gender, age, and education to include 3 AD − P subjects for each AD + P subject using a SAS macro [29] For one of the AD + P participants in the analysis, the macro only found 2 potential matches, therefore 50 AD − P subjects were included in the analyses described below. Regional ethical committees of all participating institutions approved the ADNI protocol. All study participants provided written informed consent.

### PET acquisition and processing

[^18^F]-AV1451 PET imaging was performed as part of ADNI3 project. We selected the baseline [^18^F]-AV1451 PET scan (first scan acquired) for each subject who was include in the analysis. Participants were injected with 370 MBq (10 mCi) of tracer and underwent a 30 min dynamic scan consisting of six 5-min frames 75 min post-injection. Pre-processing of PET images in ADNI (http://adni.loni.usc.edu/methods/pet-analysis-method/petanalysis/) follows four steps: (1) separate frames were co-registered to one another lessening the effects of patient motion; (2) the 5 frames were averaged to obtain a single 30 min PET image set; (3) PET images were then reoriented into a standardized space and intensity normalized; (4) Finally PET images were filtered with a scanner-specific filter function to produce images of a uniform isotropic resolution of 8 mm full with at half maximum (FWHM). For all analyses we used standardized uptake value ratio (SUVR) data normalized to whole cerebellum uptake. In order to ascertain Aβ positivity, we used Florbetapir ([^18^F]-AV45) and [^18^F]-Florbetaben data provided by ADNI. [^18^F]-AV45 PET images were acquired for 20 min (4 × 5 min frames) 50 min post-injection of 370 MBq (10.0 mCi) of tracer. [^18^F]-Florbetaben PET images were acquired for 20 min (4 × 5 min frames) 90 min post-injection of 300 MBq (8.1 mCi) of tracer. [^18^F]-AV45 and [^18^F]-Florbetaben PET images were processed following the same algorithm described above. The Aβ PET scan closest in time to the [^18^F]-AV1451 tau PET scan was used to determine Aβ positivity.

### MRI acquisition and processing

The MRI closest to the date of the [^18^F]-AV1451 PET was downloaded from ADNI for co-registration with the PET image. In ADNI 3 protocol (tau PET) [30], MRI were acquired in 3 T scanners using a high-resolution T1-weighted magnetization prepared rapid gradient echo (MPRAGE) sequence with the following parameters: TR = 2300, TE = minimum full echo, TI = 900, FOV = 208 × 240 × 256 mm, and 1 × 1 × 1 mm resolution s (detailed scan protocols can be found on https://adni.loni.usc.edu/wp-content/uploads/2017/07/ADNI3-MRI-protocols.pdf). Structural images were processed using FreeSurfer version 6.0 (http://surfer.nmr.mgh.harvard.edu/). FreeSurfer was used to extract anatomical Regions of Interest (ROI) for multiple brain regions on the native space MRI (co-registered to the PET scans). FreeSurfer segmentations were checked and manually fixed in case of inaccuracy (I.e., pial surface precluding to grey matter and white matter being erroneously assigned to grey matter). A high-resolution segmentation was also extracted to be used with the geometric transfer matrix for performing PET partial volume correction (PVC).

### Voxelwise [^18^F]-AV1451 PET data processing

To explore the differences in [^18^F]-AV1451 uptake between AD + P and AD − P, we first obtained individual voxelwise PET maps using the Mueller–Gaertner method implemented in PETSurfer (https://surfer.nmr.mgh.harvard.edu/fswiki/PetSurfer), consisting in two stages (1) orthogonalization to subtract non grey matter (GM) tissue; and (2) rescaling of each voxel divided by the fraction of GM in that voxel, that was set up in our analysis to 0.01, requiring that a particular voxel have at least 1% GM to be rescaled, otherwise the voxel is set to 0 [31, 32].

### Braak staging classification of [^18^F]-AV1451 PET and ROI analysis

From the Freesurfer segmented and parcellated structural MRIs, we created bilateral ROIs that anatomically approximated the Braak and Braak classification for AD-related tau pathology staging [15]. These non-overlapping Braak stage regions were divided into: (1) Braak I/II corresponding to the transentorhinal stage; (2) Braak III/IV corresponding to the limbic stage; and (3) Braak V/VI corresponding to the isocortical stage (see Supplemental Table S1 for a list of all the ROIs included in each Braak stage). Braak ROI-derived [^18^F]-AV1451 uptake values were averaged combining all the regions included in each stage. We also performed a partial volume correction (PVC) using the symmetric geometric transfer matrix (SGTM) to minimize PVE (radiotracer spilling out from adjacent regions) as implemented in PETSurfer [33–35]. For this, a design matrix is computed as follows. For each ROI, an image was created in the PET native space where the value in a voxel was the tissue fraction (TF) for that ROI in that voxel; this accounts for the tissue fraction effect (TFE), that is a type of partial volume effect (PVE) that occurs when multiple tissue types or ROIs occupy the same PET voxel. This image was then smoothed by the point-spread function (PSF), that in our analysis was set up at 5 FWHM. The result, the regional spread function (RSF), was then reshaped into a vector. This was repeated for each ROI (full head model) including extra-cerebral structures (structures that are neither GM nor WM).

### Neuropsychological examination

The present study examined cognitive functioning by analyzing longitudinal assessments administered as part of the ADNI protocol. The following assessments were included in the analysis. The Clinical Dementia Rating Scale Sum of Boxes (CDR-SB) [36], a semi-structured interview conducted with both the subject and informant, assess global functioning. During the task, information is gathered regarding memory, orientation, judgment, and problem-solving abilities, as well as ability to participate in community affairs, home and hobbies, and personal care. The mini-mental state exam (MMSE) [37] is a cognitive screen used to aid in detection of cognitive impairment.

### Statistical analysis

Differences in [^18^F]-AV1451 PET uptake between AD + P and AD − P groups were compared using both voxel-wise and ROI analyses. In the voxel-wise analysis (described above), cluster-wise correction for multiple comparisons was performed using a permutation simulation [38] of 5000 iterations, with a vertex-wise cluster-forming threshold of *p* < 0.0001, absolute/unassigned effect (two-sided statistical test), a cluster-wise *p* value of *p* < 0.05, and an additional Bonferroni correction of the *p* values by 3 (that is left/right hemispheres and subcortical structures).

[^18^F]-AV1451 PET uptake on Braak-derived ROIs were compared between groups using general linear models (GLM) with each Braak-derived ROI as outcome and AD group (AD + P vs. AD − P) as independent predictor. Statistical tests were two-sided, with significance determined at *p* < 0.05 corrected for multiple comparisons using false discovery rate (FDR) method. Effect sizes for differences between groups in these analyses were evaluated with Hedges and Olkin´s correction approach [39].

To assess change in cognition over time, annualized rate of change (ARC) in the respective cognitive task were generated using a linear mixed effects model. Random effects included inter-individual intercept and time (years) between neuropsychological examinations following an unstructured covariance pattern. Between-group ARC contrasts were performed with GLM as described above.

All statistical analyses were performed using SAS Studio 9.04.01 (SAS Institute Inc., Cary, NC, USA). The variance between the groups compared in the variables of interest was similar.

## Results

### Participants

Demographic and clinical characteristics of the participants are provided in Table 1. In brief, 60% AD − P participants and 58.8% of AD + P participants were male, with a mean age of 78.72 years-old (SD = 6.20) in the case of AD − P, and 79.23 years-old (SD = 6.71) in the case of AD + P.

**Table 1:** Demographic and clinical characteristics.

	AD − P	AD + P	Statistical test
*N*	50	17	
Age (years)	78.72 (6.20)	79.23 (6.71)	*t*_65_ = −0.29/*p* = 0.77
Sex (M/F)	30/20	10/7	Chi square = 0.01/*p* = 0.93
Education (years)	16.14 (2.69)	15.47 (2.53)	*t*_65_ = 0.90/*p* = 0.37
APOE (allele combination)	E2/E3 = 1	E2/E3 = 0	Chi Square = 0.77/*p* = 0.94
	E2/E4 = 2	E2/E4 = 1	
	E3/E3 = 21	E3/E3 = 6	
	E3/E4 = 18	E3/E4 = 7	
	E4/E4 = 7	E4/E4 = 3	
MMSE	26.42 (3.13)	20.94 (5.01)	*t*_65_ = 5.30/*p* < 0.0001
CDR-SB	2.65 (1.97)	6.29 (2.89)	*t*_65_ = −5.81/*p* < 0.0001

All values are mean (SD) unless stated otherwise.

*APOE* Apolipoprotein E, *MMSE* Mini Mental State Examination, *CDR-S*B Clinical Dementia Rating Sum of Boxes, *AD* *−* *P* Alzheimer’s disease without psychotic symptoms, *AD* *+* *P* Alzheimer’s disease with psychotic symptoms.

Mean years of education was 16.14 (SD = 2.69) for AD − P, and 15.47 (SD = 2.53) for AD + P. As has previously been reported, AD + P subjects were significantly more cognitively impaired even at baseline compared to matched AD − P patients [40]. APOE allele combination distribution was comparable between the two groups, with 58% APOE E4 carriers in the AD + P group and 50% in the AD − P group. The cognitive profile of AD + P subjects revealed specific impairments in episodic memory (except for delayed verbal list learning), and in executive function (Table S2). All patients in both AD + P and AD − P groups were amyloid-beta (Aβ) positive as indicated by the Aβ PET scan closest in time to the [^18^F]-AV1451 PET scan: 38 patients with [^18^F]-AV45 and 12 with [^18^F]-Florbetaben in the AD − P subgroup; and 14 patients with [^18^F]-AV45 and 3 with [^18^F]-Florbetaben in the AD + P subgroup.

### Spatial distribution of [^18^F]-AV1451 within groups

**Fig. 1:**
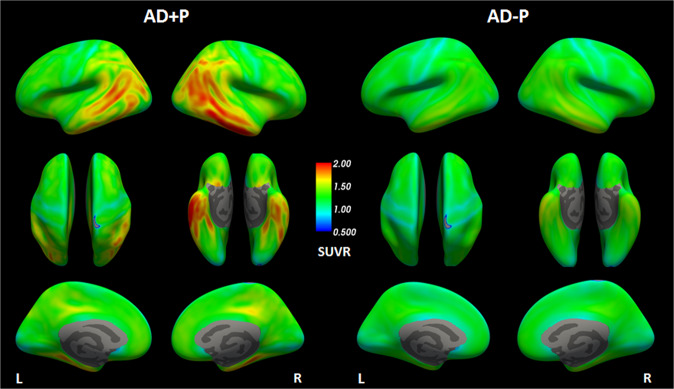
^18^F-AV-1451 SUVR uptake for AD + P and AD − P groups. Cortical uptake of ^18^F-AV-1451 tracer in the form of SUVR referenced to whole cerebellum cortex for each group in the study: AD + P and AD − P. Top row displays lateral planes of the brain, middle row superior and inferior planes, and bottom row medial planes. SUVR standardized uptake value ratio, AD + P Alzheimer´s disease with Psychotic symptoms, AD − P Alzheimer´s disease without Psychotic symptoms.

AD + P participants showed increases in tau SUVR uptake especially localized in parietal (inferior and superior parietal, precuneus and supramarginal) and temporal regions, both lateral (middle and inferior temporal) and medial temporal (fusiform gyrus, parahippocampal gyrus and entorhinal cortex); AD + P patients also showed high tau binding in medial and lateral orbital frontal regions. AD − P patients primarily showed the highest tau uptake in lateral and medial temporal lobe regions (Fig. 1).

### Voxel-wise contrast of [^18^F]-AV1451 uptake between groups

**Fig. 2:**
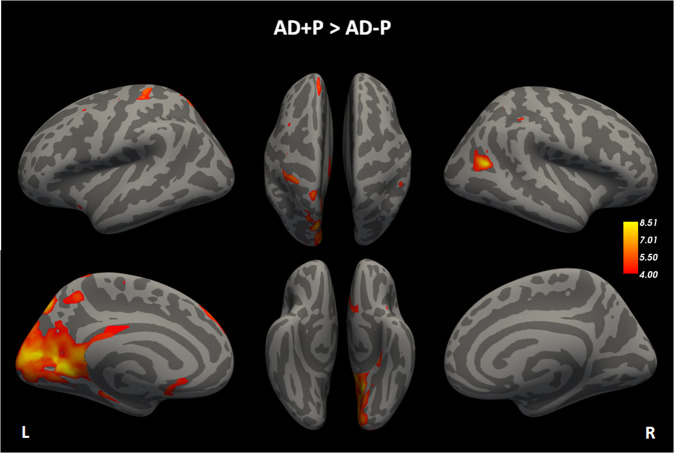
Voxelwise contrast of ^18^F-AV-1451 SUVR uptake between AD + P and AD − P groups. Contrast voxel-wise maps shows brain regions where AD + P had higher ^18^F-AV-1451 uptake compared to AD − P. Color wheel represents log10 of *p* level with threshold *p* < 10^−4^ = 0.0001. Results from multiple comparisons correction using permutations tests (see the “Methods” section) are reported in Supplementary Table 2. AD + P Alzheimer´s disease with Psychotic symptoms, AD − P Alzheimer´s disease without Psychotic symptoms.

Contrast maps between AD + P and AD − P of voxel-wise analysis revealed that mean tau cortical uptake was elevated in the AD + P group in parietal, medial temporal, posterior-cingulate/precuneus and frontal regions (Fig. 2). Cluster-wise correction for multiple comparisons provided 7 clusters in the left hemisphere comprising lingual gyrus, precuneus, postcentral gyrus, superior parietal, superior frontal, medial orbito-frontal, parahippocampus gyrus and superior parietal; Only 1 cluster in the right hemisphere comprising inferior parietal region survived the correction. Regarding subcortical volumes, left white matter volume showed higher ^18^F-AV-1451 tracer retention in AD + P compared to AD − P. A list of all the clusters that survived multiple comparisons correction are listed in Table S3 in supplement.

### ROI analyses of ^18^F-AV-1451 uptake

**Fig. 3:**
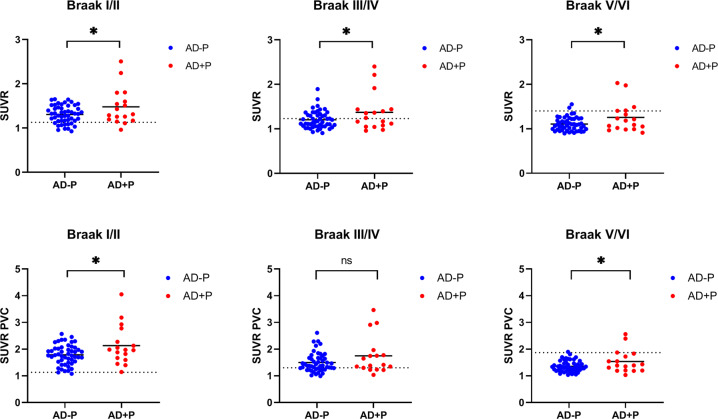
Braak stage-derived ROI analysis of ^18^F-AV-1451 uptake between AD + P and AD − P groups. Figures shows the comparisons in SUVR units referenced to whole cerebellum cortex between AD + P and AD − P. Top row display the GLM results uncorrected for PVE, and bottom row displays the results corrected for PVEs. Statistical contrasts corrected for multiple comparisons using FDR. *p < 0.05, n.s. not significant. Dashed lines represent the Braak-derived stage thresholds as provided in reference [66]. SUVR standardized uptake value ratio, AD + P Alzheimer´s disease with Psychotic symptoms, AD − P Alzheimer´s disease without Psychotic symptoms, GLM general linear model, PVE partial volume effects, FDR false discovery rate. .

ROI-based analysis using Braak stage areas of tau aggregation is shown in Fig. 3. Bilateral composites for Freesurfer-derived ROIs were averaged according to the staging of tau neuropathology proposed by Braak, resulting in anatomically distinct brain areas comprising transenthorinal (Braak stage I/II), limbic (Braak stage III/IV), and isocortical (Braak stage V/VI) regions. Figure 3 top row represents the comparison between AD + P and AD − P groups uncorrected for partial volume effects (PVE), where AD + P showed significantly higher SUVRs in all three Braak-derived ROIs composites compared to AD −P (FDR corrected *p* < 0.05). Results using PVE corrected SUVRs (Fig. 3 bottom row) showed a similar pattern except for regions included in the Braak stage III/IV, where both groups showed similar mean tau uptake. Effect sizes of the differences between AD + P and AD − P in each of these Braak-derived ROIs, revealed differences of magnitude of 0.60 for Braak I/II, 0.61 for Braak III/IV, and 0.68 for Braak V/VI for the PVE uncorrected SUVRs. PVE corrected SUVRs revealed effect sizes of 0.66 for Braak I/II, 0.50 for Braak III/IV, and 0.65 for Braak V/VI.

### Relationships between tau and cognition in AD with psychosis (AD + P)

**Fig. 4:**
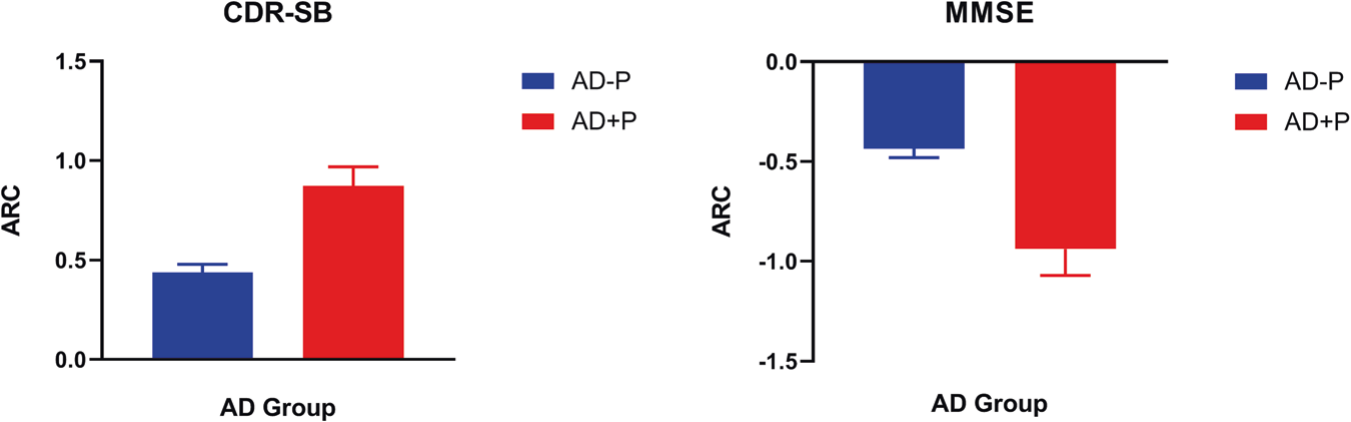
Cognitive trajectories by group. This figure shows the mean annualized rate change (ARC) in cognition by group (AD − P vs. AD + P). **A** Shows the mean ARC for CDR-SB for each group; note that for the case of CDR-SB, higher scores correspond to greater longitudinal functional impairment. **B** Shows the mean ARC for MMSE for each group; note that for the case of MMSE, lower scores correspond to greater cognitive decline. Error bars represent the standard error of the mean (SEM). AD − P Alzheimer´s disease without Psychotic symptoms, AD + P Alzheimer´s disease with Psychotic symptoms, CDR-SB Clinical Dementia Rating–Sum of Boxes, MMSE Mini Mental State Examination, ARC annualized rate change.

We calculated individual longitudinal trajectories of cognitive performance using all available assessments throughout all phases of ADNI to compare ARC between AD + P and AD − P groups. Figure 4 shows CDR-SB (Fig. 4A) and MMSE (Fig. 4B) ARC for each group. Differences between groups in the ARC were significantly different for both CDR-SB (*F*_1,65_ = 24.27/*p* < 0.0001) and MMSE (*F*_1,65_ = 21.60/*p* < 0.0001). This suggests that AD + P subjects declined more rapidly both in terms of the global measure of cognitive integrity (MMSE) and functional impairment (CDR). Spaghetti plots for individual longitudinal trajectories are reported in the supplement (Fig. S1).

## Discussion

ADNI participants who became psychotic over the course of their participation in the study evidenced markedly increased ^18^F-AV-1451 retention on PET imaging indicating a greater burden of paired helical filament tau pathology at baseline. Though primarily focused in the left hemisphere, there were relative increases in tau in frontal, temporal, and occipital cortices (Fig. 1). The differences in tau retention remained significantly greater in those who would become psychotic within all three Braak-derived ROIs (transentorhinal, limbic, and isocortical) suggesting that in addition to any particular regional localization, psychosis may be associated with a more aggressive spread of tau pathology (Fig. 3). These data are consistent with data from early post-mortem neuropathological studies in which an abundance of tau-derived neurofibrillary tangles rather than amyloid plaques were found to be a correlate of psychosis in AD [18–20, 41]. More recent immunohistochemical and biochemical studies done in our laboratory and elsewhere focusing on the frontal cortex, have found that the tau in the brains of those with AD who suffered with psychosis is hyperphosphorylated relative to those who did not, significant as tau phosphorylation is widely believed to be a critical event in the formation of neurofibrillary tangles [21, 22]. Though no previously published studies have investigated the relationship between tau pathology captured with functional neuroimaging and AD psychosis, previous studies have associated increased tau PET ligand retention with psychosis in other conditions such as late-life depression [42], and psychosis in traumatic brain injury [43]. Taken together, these data suggest that tau pathology may be uniquely psychotogenic in neuropsychiatric conditions.

In addition to an increase in tau pathology, those who became psychotic over the course of participation in ADNI experienced a more precipitous and highly significant global cognitive and functional decline as evidenced by the differences in the slopes of the trajectories of MMSE and CDR over time when compared with those who were not psychotic (Fig. 4). Previous studies have found that those who experience psychosis over the course of AD experience a more rapid decline in cognition, to the extent that this acceleration has been identified as a risk factor for the development of psychosis [44]. Even in early stages of disease (MCI), evidence suggests that those who eventually become psychotic are more cognitively impaired [40]. The increase in the velocity of cognitive decline on a variety of measures in psychotic patients with AD has been well replicated [6, 45, 46]. In an earlier analysis of ADNI participants (without overlap in the current analysis), we reported that the velocity of change over time in MMSE scores and functional performance assessment distinguishes the course of illness in psychotic AD from non-psychotic AD [8]. Additionally, we have previously reported on specific relationships between particular domains of cognitive function and psychosis in AD, including impairment in working memory [7, 8] and social cognition [47]. In a recent ADNI analysis exploring the relationship between cognitive rating scales and the catalogue of neurobehavioral variables assessed with the NPI to determine which specific symptoms correlate most closely with cognitive dysfunction in AD, it was reported that psychotic symptoms had the strongest correlation with cognitive impairment [28]. The current study suggests that the aggressivity of tau pathology in psychosis may be the driver of the cognitive morbidity so consistently reported.

The identification of a relative abundance of tau in particular brain regions in those who would eventually develop psychosis in the current report may be phenomenologically relevant for psychotic and cognitive phenotypes of AD. The lingual gyrus, also referred to as medial occipitotemporal gyrus, is a structure in the visual cortex whose activation has been associated with non-verbal logic-rooted conditions and the processing of emotional images. For example, in a study designed to ascertain the participation of a variety of brain regions in theory-of-mind skills and the attribution of intentions to others utilizing the interpretation of comic strips together PET neuroimaging, the activation of the lingual gyri differentiated physical logic in the context of the presentation of human characters from physical logic related to non-human objects [48]. In another study utilizing fMRI, the emotional valence of responses to visual imagery depended in part on the activation of the lingual gyrus [49]. In the current report the left lingual gyrus was found to have increased ^18^F-AV-1451 retention in those who would become psychotic over the course of the study. The fusiform gyrus, another structure in the visual cortex that evidenced high uptake of ^18^F-AV-1451 in psychotic AD, has been implicated in the processing of information related to faces [50], and has been shown to receive direct inputs from the lingual gyrus [51]. The fusiform gyrus is believed to play a key role in affective facial processing [52]. An abundance of tau pathology in these regions may explain, in part, the recent association we observed between impaired facial affective processing and the development of psychosis in AD [47]. In that study, subjects with AD were followed longitudinally and assessed for the ability to identify affect from pictures of faces. Over the course of the study, those who became psychotic had significant impairment in facial affect recognition compared with those who did not [47]. Increased tau pathology in visual cortex including the fusiform and lingual gyri may impair facial affective processing and increase the risk of misattributing intention to behavior, leading to psychosis. The increased tau pathology evidenced in the superior fontal and medial orbitofrontal regions of those with psychosis is consistent with the neuropathologic studies alluded to above [18–22, 41], including one controlled for Braak staging [22]. Earlier reports that have attempted to elucidate localization of neurodegeneration in AD-related psychosis from FDG-PET investigations have found evidence of decreased metabolism in the frontal cortex [53–55]. In our own previous ADNI report employing FDG-PET ROI methodology (that did not include posterior regions), we observed a relationship between the development of psychotic symptoms and orbitofrontal hypometabolism [8]. Although the current ADNI report does not overlap in subjects with the previous report, larger burdens of tau pathology in frontal regions may explain previous FDG-PET findings. The increased retention of tau PET ligand in medial temporal structures of psychotic subjects who declined more rapidly in the current report is consistent with previously reported correlations between cognitive impairment and ^18^F-AV-1451 retention. In an ADNI analysis designed in part to evaluate the relationship between MMSE decline and regional ^18^F-AV-1451 uptake, declining MMSE was associated with^18^F-AV-1451 binding in the amygdala, entorhinal cortex, parahippocampus, and fusiform [26].

There are several limitations in the current study. We did not match participants who became psychotic with those who did not over the course of the study on baseline measures of cognition. It could be argued that the increased burden of cognitive impairment in psychosis is related to disease severity, and that to isolate tau in a unique association with psychosis would require controlling for disease severity with markers of cognition. However, as the topography of neurofibrillary tangle pathology has been closely correlated with the severity of dementia [14, 56–60], controlling for disease severity would in essence be controlling for tau. Additionally, as psychosis has also been reported to be associated with a more rapid decline even in very early phases of the illness [61], without exact onset-of-illness data a failure to find a difference in tau pathology between the cognitively matched groups could obscure a more rapid accrual of tau pathology in the psychotic participants. For these reasons we chose not to match for disease severity, and followed cognition in order to determine whether an accelerated decline distinguished the groups. Future studies that focus on longitudinal assessments of tau aggregation with tau imaging in psychotic and non-psychotic patients that begin from disease-onset would help to clarify the relationship between the increase in tau deposition observed in the current report and the accelerated trajectory of cognitive decline. Further, as previous ADNI investigators have pointed out, utilizing the NPI-Q is not the most sensitive way to diagnose psychotic symptoms [28]. The interrogation of fixed false beliefs that comprise delusions relies on the opening question “Does the patient have false beliefs such as thinking that others are stealing from him/her or planning to harm him/her in some way?”, while hallucinations are elicited by the questions “Does the patient have hallucinations such as false visions or voices? Does he or she seem to hear or see things that are not present?” [62] It may be that psychosis was missed in some of the subjects who were psychotic. In some ways, that would increase the power of the current study, in that even with the limitation of the sensitivity of this time-bound assessment, subjects who were found to have psychotic symptoms significantly diverged from those who were not. Due to limited sample size and power, we were unable to confidently evaluate the effects of different psychosis phenotypes, the effects of gender, and the effects of race/ethnicity that may affect the specificity and generalizability of our results. It may be that particular topographical distributions of tau pathology contribute separately to the development of delusions or delusional subtypes and hallucinations, as it has been found that these correlate independently with the velocity of cognitive decline associated with psychosis [63]. We were unable to control for the use of medications, such as antipsychotic medications, that could increase the rate of cognitive decline in those with psychosis [64]. While antipsychotic medications could have an impact on tau pathology, work done in our laboratory suggests that antipsychotics reduce, rather than increase, tau phosphorylation, suggesting that dopamine blockade could prevent paired helical filament formation [65]. Finally, our voxel-wise analysis performed a final Bonferroni correction for tissue type (left and right hemispheres and subcortical structures). This statistical correction of the p level is the default method employed by Freesurfer to correct for the three types of independent analysis the pipeline performs. This rather conservative multiple comparisons correction method, may have been too restrictive in our voxel-wise analysis limiting the amount of voxels surviving the adjustment for statistical significance.

In conclusion, these findings suggest that psychosis in Alzheimer’s is a rapidly progressive clinical subtype driven by an aggressive tauopathy affecting multiple brain structures including those that support social cognition. Further research is required to determine whether identification of tau pathology in these regions may be predictive in prospective studies, and whether anti-tau therapies such as monoclonal antibodies to tau now being investigated in clinical trials may have a place in the antipsychotic treatment armamentarium.

## Supplementary information

Supplemental Information
