# Supplementary material for: Increased retention of tau PET ligand [18F]-AV1451 in Alzheimer’s Disease Psychosis
Source: Transl Psychiatry. 2022 Feb 26;12:82. doi: 10.1038/s41398-022-01850-z (PMC8881582; doi:10.1038/s41398-022-01850-z)
Supplement: Supplementary file 1 — Supplemental Information [file 41398_2022_1850_MOESM1_ESM.pdf]

SUPPLEMENTAL METHODS

MRI Freesurfer processing

Cortical reconstruction and volumetric segmentation was performed with the Freesurfer image analysis suite, which is documented and freely available for download online (http://surfer.nmr.mgh.harvard.edu/). The technical details of these procedures are described in prior publications [1-14]. Briefly, this processing includes motion correction and averaging [13] of multiple volumetric T1 weighted images (when more than one is available), removal of non-brain tissue using a hybrid watershed/surface deformation procedure [12], automated Talairach transformation, segmentation of the subcortical white matter and deep gray matter volumetric structures (including hippocampus, amygdala, caudate, putamen, ventricles) [5, 6] intensity normalization [15], tessellation of the gray matter white matter boundary, automated topology correction [4, 16], and surface deformation following intensity gradients to optimally place the gray/white and gray/cerebrospinal fluid borders at the location where the greatest shift in intensity defines the transition to the other tissue class [1-3]. Once the cortical models are complete, a number of deformable procedures can be performed for further data processing and analysis including surface inflation [7], registration to a spherical atlas which is based on individual cortical folding patterns to match cortical geometry across subjects [8], parcellation of the cerebral cortex into units with respect to gyral and sulcal structure [9, 17], and creation of a variety of surface based data including maps of curvature and sulcal depth. This method uses both intensity and continuity information from the entire three dimensional MR volume in segmentation and deformation procedures to produce representations of cortical thickness, calculated as the closest distance from the gray/white boundary to the gray/CSF boundary at each vertex on the tessellated surface [3]. The maps are created using spatial intensity gradients across tissue classes and are therefore not simply reliant on absolute signal intensity. The maps produced are not restricted to the voxel resolution of the original data thus are capable of detecting submillimeter differences between groups. Procedures for the measurement of cortical thickness have been validated against histological analysis [18] and manual measurements [19, 20]. Freesurfer morphometric procedures have been demonstrated to show good test-retest reliability across scanner manufacturers and across field strengths [10, 14].

SUPPLEMENTAL TABLES AND FIGURES

Table S1. List of ROIs included in the calculation of the Braak-derived stages of AD-related tau neuropathology.

Braak Stage	FreeSurfer ROIs averaged for both left and right hemispheres
**Braak I/II**	Entorhinal & Hippocampus
**Braak III/IV**	Parahippocampal gyrus, Fusiform gyrus, Lingual gyrus, Amygdala, Inferior Temporal, Middle Temporal, Temporal Pole Caudal Anterior Cingulate, Rostral Anterior Cingulate, Posterior Cingulate, Isthmus Cingulate, and Insula
**Braak V/VI**	Superior Frontal, Lateral Orbitofrontal, Medial Orbitofrontal, Frontal Pole, Caudal Middlefrontal, Rostral Middlefrontal, Pars Opercularis, Pars Orbitalis, Pars Triangularis, Lateral Occipital, Supramarginal, Inferior Parietal, Superior Parietal, Precuneus, Superior Temporal, Banks of Superior Temporal Sulcus, Transverse Temporal, Pericalcarine, Cuneus, Postcentral gyrus, Precentral gyrus, and Paracentral gyrus

Table S2. Cognitive profile of AD-P and AD+P groups.

	AD-P	AD+P	Statistical Test and p level
**N**	50	17	N/A
**Logical Memory Immediate**	8.32 (4.39)	3.00 (2.89)	t_{65}= 4.66 / p< 0.0001
**Logical Memory Delayed**	5.62 (4.55)	1.176 (2.16)	t_{65}= 3.86 / p= 0.0003
**AVLT Delayed 30 minutes**	1.82 (2.69)	0.71 (1.53)	t_{65}= 1.61 / p= 0.11
**AVLT Delayed Total**	9.14 (4.02)	4.71 (3.50)	t_{65}= 4.05 / p= 0.0001
**Category Fluency Animals**	15.60 (5.24)	11.53 (6.06)	t_{65}= 2.66 / p= 0.01
**TMT-A**	43.80 (20.34)	66.06 (33.81)[a]	t_{64}= -3.21 / p= 0.002
**TMT-B**	140.90 (83.15)[a]	231.30 (94.95)[b]	t_{62}= -3.56 / p= 0.0007

All values are mean (SD) unless stated otherwise. AD-P = Alzheimer's disease without psychotic symptoms; AD+P = Alzheimer's disease with psychotic symptoms; AVLT = Auditory Verbal Learning Test; TMT= Trail Making Test; [a] 1 missing observation; [b] 2 missing observations.

Table S3. Cluster-wise correction for multiple comparisons (permutation tests). List of ROIs that survived multiple comparisons corrections in the voxelwise contrast for AD+P versus AD-P. AD-P = Alzheimer's disease without psychotic symptoms; AD+P = Alzheimer's disease with psychotic symptoms; Max = maximum -log10 (p value) in the cluster; VtxMax = vertex number at the maximum; Size = surface area (mm^2) of cluster; MNIX, MNIY, and MNIZ is the talairach (MNI305) coordinate of the maximum; CWP = clusterwise p-value, this is the p-value of the cluster; CWP 90% CI = CWPLow and CWPHi 90% confidence interval for CWP.

LEFT HEMISPHERE	MAX	VTXMAX	SIZE(MM^2)	MNIX	MNIY	MNIZ	CWP	CWP 90% CI
**Lingual**	8.510	71897	6311.13	-25.8	-61.4	1.9	0.0006	0 – 0.0012
**Precuneus**	7.745	92684	452.6	-8.5	-73.9	44.9	0.00718	0.00479 – 0.01017
**Postcentral**	5.839	121275	222.82	-24.3	-31.2	70.1	0.01493	0.01136 – 0.01848
**Precuneus**	5.386	35531	193.33	-8.3	-49.4	50.9	0.01789	0.01374 – 0.02204
**Superior Parietal**	5.578	150723	185.77	-19.1	-65	58.5	0.01848	0.01433 – 0.02263
**Superior Frontal**	5.578	58356	177.34	-6.9	45	44.2	0.01848	0.01433 – 0.02263
**Medial Orbito-Frontal**	5.071	71602	143.51	-5	7	-8.4	0.02381	0.01908 – 0.02852
**Parahippocampal**	5.707	92049	134.25	-23.1	-28.1	-16.6	0.02558	0.02085 – 0.03029
**Superior Parietal**	5.953	140336	112.82	-16.7	-43.9	73.1	0.0297	0.0244 – 0.03498

RIGHT HEMISPHERE	MAX	VTXMAX	SIZE(MM^2)	MNIX	MNIY	MNIZ	CWP	CWP 90% CI
**Inferior Parietal**	5.388	87460	303.67	42.0	-57.1	11.0	0.01255	0.00897 - 0.01611

SUBCORTICAL ROI	MAX	SIZE(MM^3)	MNIX	MNIY	MNIZ	CWP	CWP 90% CI
**Left White Matter**	5.829	1392.0	-24	-13	-31	0.00718	0.00479 - 0.01017

Table S4. Distribution of participants below and above the Braak-derived stage thresholds. The table shows the number of participants below and above the SUVR cut-off levels for both AD-P and AD+P groups uncorrected and corrected for partial volume effects (PVE). Regarding the AD+P group, 88.2% in Braak I/II stage, 52.9% in Braak III/VI stage, and 23.5% in Braak V/VI stage were above the cut-off point in the uncorrected results; 100% in Braak I/II stage, 70.6% in Braak III/VI stage, and 17.6% in Braak V/VI stage were above the cut-off point in the partial volume corrected (PVC) results. These cut-offs were obtained using two samples, one consisting in 12 young and middle-aged controls (age range 20-60 years old), 74 older cognitively normal controls (age range 61-93 years old), 12 mild cognitive impairment (MCI) participants, and 48 Alzheimer's disease (AD) participants from the University of California San Francisco (UCSF); the other sample from ADNI database consisted in 42 healthy controls (65-83 years old), 19 MCIs and 9 AD patients [21]. SUVR = Standardized Uptake Value Ratio; AD-P = Alzheimer's disease without psychotic symptoms; AD+P = Alzheimer's disease with psychotic symptoms.

**SUVR Uncorrected for PVE**					
**Braak I/II**		**Braak III/IV**		**Braak V/VI**	
AD-P > 1.135	AD+P > 1.135	AD-P > 1.232	AD+P > 1.232	AD-P > 1.407	AD+P > 1.407
39 (78.0%)	15 (88.2%)	17 (34.0%)	9 (52.9%)	2 (4.0%)	4 (23.5%)
**SUVR Corrected for PVE**					
**Braak I/II**		**Braak III/IV**		**Braak V/VI**	
AD-P > 1.129	AD+P > 1.129	AD-P > 1.304	AD+P > 1.304	AD-P > 1.873	AD+P > 1.873
49 (98.0%)	17 (100%)	34 (68.0%)	12 (70.6%)	1 (2.0%)	3 (17.6%)

Table S5. CDR-domain comparison between AD-P and AD+P scores. Values correspond to means and standard deviations (SD) in brackets. CDR = Clinical Dementia Rating; AD-P = Alzheimer’s disease without psychotic symptoms; AD+P = Alzheimer’s disease with psychotic symptoms.

	AD-P	AD+P	Statistical Test
**N**	50	17	
**CDR Orientation**	0.44 (0.42)	1.21 (0.75)	t_{65}= -5.37 / p< 0.0001
**CDR Memory**	0.68 (0.33)	1.32 (0.64)	t_{65}= -5.21 / p< 0.0001
**CDR Judgement/Problem Solving**	0.53 (0.38)	1.12 (0.65)	t_{65}= -4.51 / p< 0.0001
**CDR Community Affairs**	0.35 (0.43)	1.00 (0.56)	t_{65}= -4.97 / p< 0.0001
**CDR Home & Hobbies**	0.43 (0.45)	1.00 (0.47)	t_{65}= -4.45 / p< 0.0001
**CDR Personal Care**	0.22 (0.46)	0.65 (0.61)	t_{65}= -3.02 / p= 0.004

Figure S1. Spaghetti plots of cognitive scores. Patterns of individual longitudinal scores on CDR-SB and MMSE. Figure S 1A displays the scores on CDR-SB and Figure S 1B scores on MMSE. Red lines correspond to participants included in the AD+P, and blue lines to participants included in the AD-P group. CDR-SB = Clinical Dementia Rating Sum of Boxes; MMSE = Mini Mental State Examination; AD-P = Alzheimer's disease without psychotic symptoms; AD+P = Alzheimer's disease with psychotic symptoms.

S 1A **S 1B**

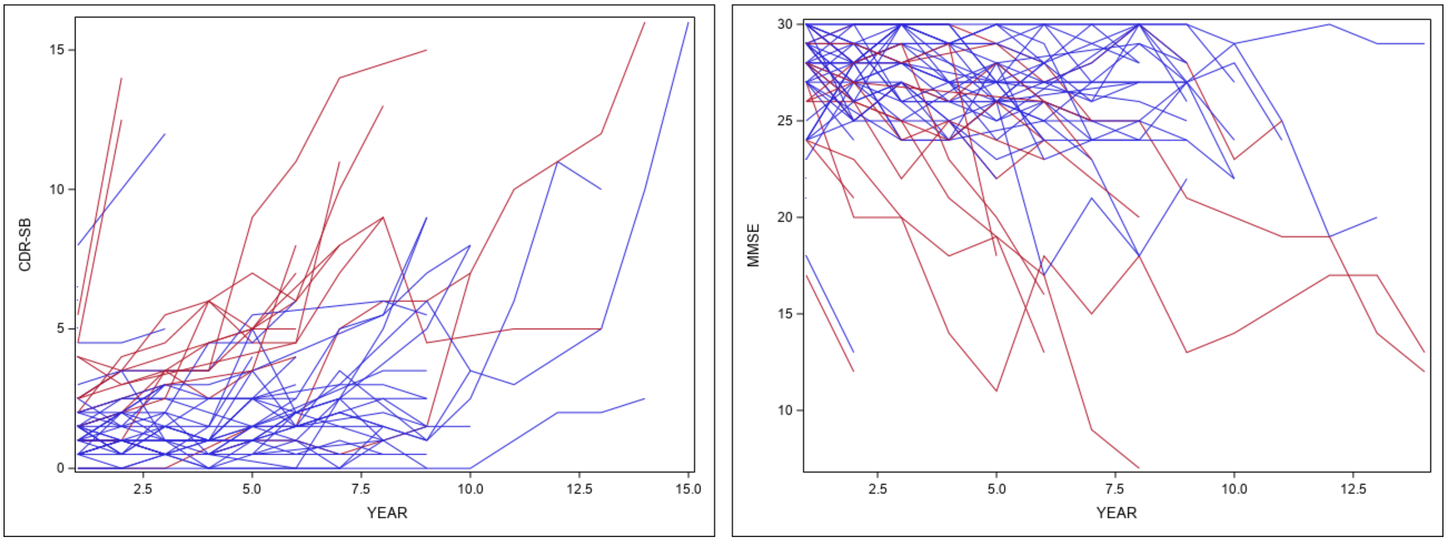

SUPPLEMENTAL REFERENCES

1. Dale, A.M., B. Fischl, and M.I. Sereno, Cortical surface-based analysis. I. Segmentation and surface reconstruction. Neuroimage, 1999. 9(2): p. 179-94.

2. Dale, A.M. and M.I. Sereno, Improved Localizadon of Cortical Activity by Combining EEG and MEG with MRI Cortical Surface Reconstruction: A Linear Approach. J Cogn Neurosci, 1993. 5(2): p. 162-76.

3. Fischl, B. and A.M. Dale, Measuring the thickness of the human cerebral cortex from magnetic resonance images. Proc Natl Acad Sci U S A, 2000. 97(20): p. 11050-5.

4. Fischl, B., A. Liu, and A.M. Dale, Automated manifold surgery: constructing geometrically accurate and topologically correct models of the human cerebral cortex. IEEE Trans Med Imaging, 2001. 20(1): p. 70-80.

5. Fischl, B., et al., Whole brain segmentation: automated labeling of neuroanatomical structures in the human brain. Neuron, 2002. 33(3): p. 341-55.

6. Fischl, B., et al., Sequence-independent segmentation of magnetic resonance images. Neuroimage, 2004. 23 Suppl 1: p. S69-84.

7. Fischl, B., M.I. Sereno, and A.M. Dale, Cortical surface-based analysis. II: Inflation, flattening, and a surface-based coordinate system. Neuroimage, 1999. 9(2): p. 195-207.

8. Fischl, B., et al., High-resolution intersubject averaging and a coordinate system for the cortical surface. Hum Brain Mapp, 1999. 8(4): p. 272-84.

9. Fischl, B., et al., Automatically parcellating the human cerebral cortex. Cereb Cortex, 2004. 14(1): p. 11-22.

10. Han, X., et al., Reliability of MRI-derived measurements of human cerebral cortical thickness: the effects of field strength, scanner upgrade and manufacturer. Neuroimage, 2006. 32(1): p. 180-94.

11. Jovicich, J., et al., Reliability in multi-site structural MRI studies: effects of gradient non-linearity correction on phantom and human data. Neuroimage, 2006. 30(2): p. 436-43.

12. Segonne, F., et al., A hybrid approach to the skull stripping problem in MRI. Neuroimage, 2004. 22(3): p. 1060-75.

13. Reuter, M., H.D. Rosas, and B. Fischl, Highly accurate inverse consistent registration: a robust approach. Neuroimage, 2010. 53(4): p. 1181-96.

14. Reuter, M., et al., Within-subject template estimation for unbiased longitudinal image analysis. Neuroimage, 2012. 61(4): p. 1402-18.

15. Sled, J.G., A.P. Zijdenbos, and A.C. Evans, A nonparametric method for automatic correction of intensity nonuniformity in MRI data. IEEE Trans Med Imaging, 1998. 17(1): p. 87-97.

16. Segonne, F., J. Pacheco, and B. Fischl, Geometrically accurate topology-correction of cortical surfaces using nonseparating loops. IEEE Trans Med Imaging, 2007. 26(4): p. 518-29.

17. Desikan, R.S., et al., An automated labeling system for subdividing the human cerebral cortex on MRI scans into gyral based regions of interest. Neuroimage, 2006. 31(3): p. 968-80.

18. Rosas, H.D., et al., Regional and progressive thinning of the cortical ribbon in Huntington's disease. Neurology, 2002. 58(5): p. 695-701.

19. Kuperberg, G.R., et al., Regionally localized thinning of the cerebral cortex in schizophrenia. Arch Gen Psychiatry, 2003. 60(9): p. 878-88.

20. Salat, D.H., et al., Thinning of the cerebral cortex in aging. Cereb Cortex, 2004. 14(7): p. 721-30.

21. Maass, A., et al., Comparison of multiple tau-PET measures as biomarkers in aging and Alzheimer's disease. Neuroimage, 2017. 157: p. 448-463.
